# Preterm Birth Affects the Risk of Developing Immune-Mediated Diseases

**DOI:** 10.3389/fimmu.2017.01266

**Published:** 2017-10-09

**Authors:** Sybelle Goedicke-Fritz, Christoph Härtel, Gabriela Krasteva-Christ, Matthias V. Kopp, Sascha Meyer, Michael Zemlin

**Affiliations:** ^1^Laboratory of Neonatology and Pediatric Immunology, Department of Pediatrics, Philipps University Marburg, Marburg, Germany; ^2^Department of General Pediatrics and Neonatology, Saarland University Medical School, Homburg, Germany; ^3^Department of Pediatrics, University of Lübeck, Lübeck, Germany; ^4^Institute of Anatomy and Cell Biology, Saarland University Medical School, Homburg, Germany; ^5^Department of Pediatric Allergy and Pulmonology, University of Lübeck, Airway Research-Center North (ARCN), Lübeck, Germany

**Keywords:** preterm neonate, allergy, atopic dermatitis, bronchial asthma, immune imprinting, microbiome, bronchitis, bronchopulmonary dysplasia

## Abstract

Prematurity affects approximately 10% of all children, resulting in drastically altered antigen exposure due to premature confrontation with microbes, nutritional antigens, and other environmental factors. During the last trimester of pregnancy, the fetal immune system adapts to tolerate maternal and self-antigens, while also preparing for postnatal immune defense by acquiring passive immunity from the mother. Since the perinatal period is regarded as the most important “window of opportunity” for imprinting metabolism and immunity, preterm birth may have long-term consequences for the development of immune-mediated diseases. Intriguingly, preterm neonates appear to develop bronchial asthma more frequently, but atopic dermatitis less frequently in comparison to term neonates. The longitudinal study of preterm neonates could offer important insights into the process of imprinting for immune-mediated diseases. On the one hand, preterm birth may interrupt influences of the intrauterine environment on the fetus that increase or decrease the risk of later immune disease (e.g., maternal antibodies and placenta-derived factors), whereas on the other hand, it may lead to the premature exposure to protective or harmful extrauterine factors such as microbiota and nutritional antigen. Solving this puzzle may help unravel new preventive and therapeutic approaches for immune diseases.

## Introduction

Almost 10% of all children are born prematurely (<37 weeks of gestation), and more than 1% of all children are born very preterm (<32 weeks) (1). Complications associated with preterm birth are the leading cause of death among infants (1). In survivors, the risk of long-term sequelae increases with decreasing gestational age (2). Due to the provision of highly sophisticated neonatal intensive care, survival rates among children born at 24 weeks of gestation are greater than 50%. The children who survive extreme prematurity will have, consequently, spent less than 60% of the normal gestation duration (37–41 weeks) *in utero* (3).

During this time, the fetal immune system is exposed to maternal and self-antigens, which should be tolerated. In addition, the fetus is exposed to environmental antigens that are transferred in a controlled manner through the placenta and into the fetal blood stream and/or into the amniotic fluid (4). Thus, the mucosa of the gastrointestinal tract (GIT) is exposed to swallowed environmental antigens that can elicit immune reactions (4), although the type and load of antigens and the fetal immune response differ quantitatively and qualitatively from postnatal immune reactions. Immediately after birth, the preterm neonate establishes a dermal and gastrointestinal microbiome, and the adaptive immune system starts to generate secondary immune responses: in the lymphoid organs, secondary lymph follicles give rise to class-switched B cells and affinity-driven maturation (5). Since the GIT mucosa of preterm neonates is permeable for macromolecules and even bacteria, the exposure to foreign antigens is not limited to the mucosal and skin surfaces; significant amounts of antigens may reach the lymph system and the blood stream (5). This accounts for the high susceptibility of preterm neonates to infection. In addition, the surface microbiome of preterm neonates differs from that of term neonates (6). Taken together, it must be expected that these dramatic changes in antigen confrontation caused by preterm birth, compared with the uninterrupted physiological intrauterine development, will have long-term effects on the immune system.

In support of this hypothesis, epidemiological studies have revealed that the incidence of immune-mediated diseases differ between preterm and term neonates. Intriguingly, preterm neonates develop atopic dermatitis less frequently (7, 8) and asthma more frequently (9, 10) than term neonates.

Genetic association studies revealed conflicting results regarding the association between atopic diseases in the mother and preterm delivery (11, 12): in one study, allergic rhinitis was less frequent among mothers of very low birthweight (VLBW) neonates (11), whereas in another study, maternal asthma was associated with preterm birth (12). It was hypothesized that a Th2 bias could protect against preterm delivery (13). Intriguingly, some factors are associated with both preterm birth and the absence of atopic disease, such as lower socioeconomic status (14, 15).

Immunological changes to the feto-maternal unit can contribute to preterm birth (12). Thus, immunological characteristics of children and adolescents born prematurely might represent a mixture of individual predispositions, which were the cause of preterm birth, as well as some acquired properties, that were the consequence of preterm birth.

A better understanding of the long-term effects of preterm birth on the immune system might give insight into new therapeutic approaches to reduce the risk of immune-mediated diseases.

## Factors That Alter Inflammatory Responses

### Premature Exposure to Extrauterine Antigens

**Figure 1:**
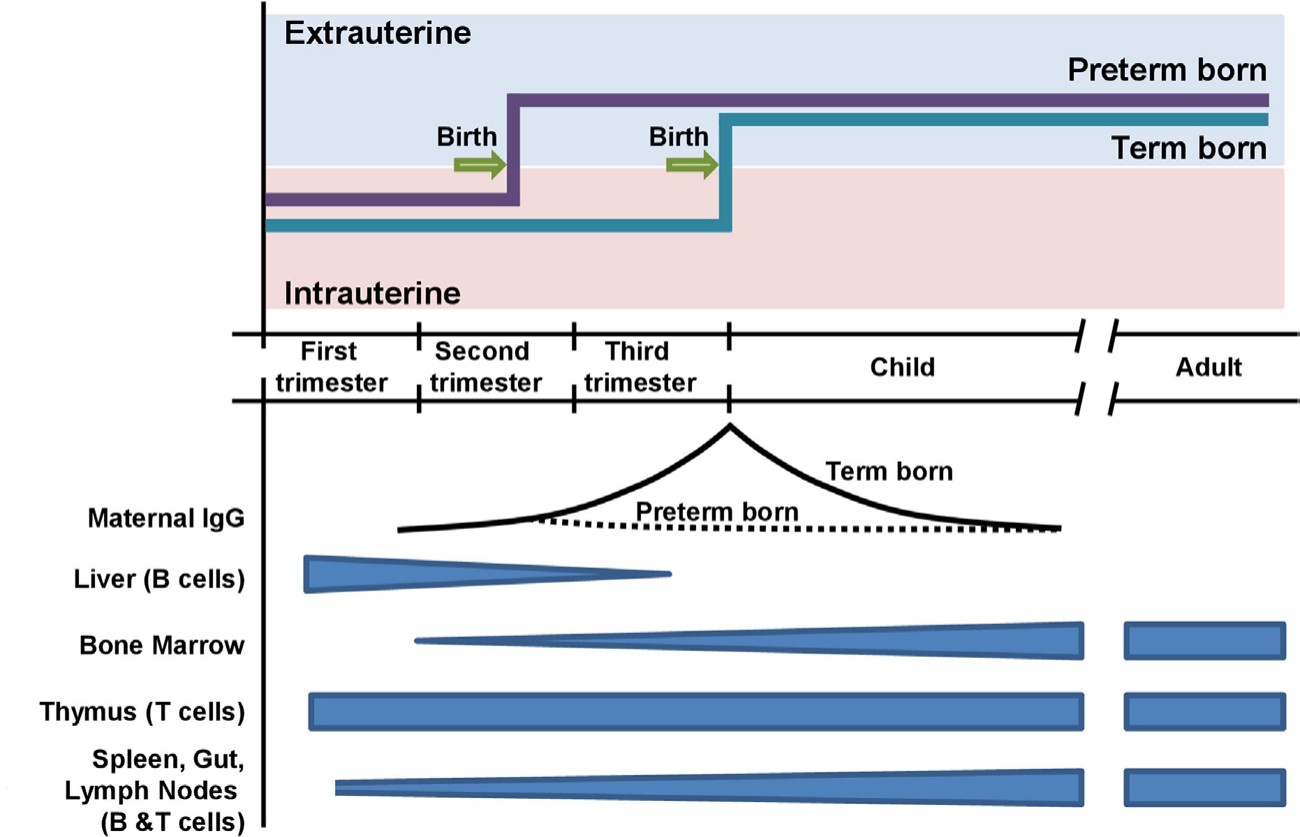
Timing of preterm and term birth in relation to the ontogeny of the adaptive immune system. Preterm neonates are exposed to extrauterine antigens before the completion of transplacental transmission of maternal IgG.

In the fetus, the immune system undergoes a controlled maturational process (Figure 1) (16). In VLBW neonates, the precursors of lymph nodes and Peyer plaques are characterized by a radially organized medulla without a B cell-rich cortex (16, 17). After birth, preterm neonates rapidly establish a repertoire of class-switched B cells, but the expressed IgG and IgA heavy-chain repertoire maintains fetal characteristics, such as short CDR-H3 regions, biased diversity gene usage, and low numbers of somatic mutations (18, 19). In congruency with these molecular features, preterm neonates produce fewer antibodies with lower antigen affinity in response to vaccination (20). The secondary antibody repertoire diversifies slower in preterm neonates than in term neonates (18). At the expected date of birth, preterm neonates express a diverse secondary antibody repertoire whereas class-switched B cells are almost absent in term newborns (18, 19). The stimulus of birth is thus the trigger for the development of secondary antibody repertoires, which is associated with the production of memory cells and plasma cells. Due to the longevity of plasma cells, one could hypothesize that this unique “fetal-like” low affinity antibody repertoire may persist for years or even decades (21, 22).

Fetal antibody production is supplemented by transmission of maternal antibodies during the last trimester. It is still under debate whether maternal antibodies confer passive immunity exclusively or whether they elicit an idiotypic network among B cell receptors and/or T cell receptors (23). In VLBW neonates, the maternal antibody concentrations have only reached approximately 10–20% of the level found in term neonates. Possibly, the reduced protection from pathogens and the diminished opportunity to mount an active response to maternal antibodies could have consequences that reach beyond the physiological duration of maternally derived passive immunity.

The development of antibody repertoires parallels the diversification of T cell receptors during ontogeny (24) and the transition from a Th2 bias toward a Th1 bias (25, 26). The hypothesis that premature antigen contact in Th2-biased preterm neonates could promote allergic sensitization was not supported by clinical observation (6, 27).

### Fetal and Postnatal Stress

Stress contributes to preterm birth, and preterm birth is associated with postnatal stress. Based on this reciprocal interaction, we hypothesize that stress has consequences on the developing immune system of preterm infants and their long-term risk of developing immune-mediated diseases. This hypothesis is derived from several aspects.

Intrauterine stress conditions (i.e., sociodemographic and obstetric risk factors, antenatal hospitalization, and nutritional abnormalities) contribute to preterm birth (28, 29). Stress can even promote epigenetic inheritance of the predisposition to preterm birth across generations (30, 31).

The association between intrauterine stress, programming fetal responses, and postnatal immunity is well established (32, 33).

The largest longitudinal human prospective study used a natural disaster—the Quebec ice storm, which left three million people without electricity for 45 days—to explore the effects of stress on the offspring of 224 women who were pregnant during the crisis. The ICESTORM project found higher rates of preterm birth in relation to the objective hardship of maternal stress (days without electricity). Notably, immunological dysregulations were found in the offspring at 13 years of age, i.e., reduced proportions of CD4+ cells and increased levels of pro-inflammatory cytokines (TNF-α, IL-1β, and IL-6) and Th2 cytokines such as IL-4 and IL-13 (34).

Preterm infants need medical support, which itself is associated with stress. The improved survival of extremely preterm infants is at the cost of over 100 invasive procedures during their hospital stay and exposure to disturbing influences such as tactile stimulation, light, and noise. The preterm infant responds to such exposures with observed changes in vital and behavioral parameters and increased metabolic demands. Early-life stress and trauma may lead to a long-term dysregulation of immune responses, i.e., sustained inflammation in adulthood (34, 35). Interestingly, these effects may be mediated by perturbations of the delicate immune–microbiota relationship (36, 37). Stress reduction, e.g., less-invasive medical treatment, cycled light, and single family rooms, reduces the length of stay in hospital and the morbidity risk (38–40).

### Premature Activation of the Chemosensory System

The premature exposure to extrauterine antigens, such as microbiota, nutrition, and medication, represents a non-physiological stimulation of all the senses, including taste and the chemosensory system. Chemosensation is a recently discovered mechanism of bacterial pathogen recognition in mammals that is conferred by classical taste receptors (T2R). Originally discovered in tongue taste buds (41, 42), T2R have been found in many extraoral tissues, including the respiratory system (43–45), especially in solitary chemosensory cells, brush cells, ciliated cells, and smooth muscle cells (46). In the upper and lower airways, detection of bitter molecules secreted by bacteria evokes innate immune responses to clear the airways of pathogens (46–49).

The developmental trajectory of chemosensory cells in the respiratory tract and other tissues is unknown (50). The number of chemosensory cells increases upon damage or stimulation of the airway epithelium (51, 52). Flavors from the mother’s diet are transmitted to the offspring through the amniotic fluid and breast milk. After 6 months of gestation, the amniotic fluid is also inhaled resulting in likely the first chemosensory experience in the lungs (53). Since taste signals undergo dynamic changes in the fetus and newborn, we hypothesize that exposure to nutritional antigens after preterm birth may influence the development of the chemosensory system, potentially with long-term effects. Moreover, an immature chemosensory system might contribute to the altered immune responses in the preterm neonate.

It is currently unclear how the normal, “healthy” microbiota influence the development and function of the chemosensory system in the respiratory tract. Many medications commonly given to preterm neonates, e.g., antibiotics, alter the classical chemosenses, taste, and smell (53, 54). In addition, preterm neonates are exposed to various olfactory and gustatory stimuli such as disinfectants and gastroesophageal reflux. All these factors might lead to an inadequate stimulation of the chemosensory system in preterm neonates. Future studies should clarify whether the premature exposure with extrauterine stimuli alters the maturation of the chemosensory system and/or the mucosal immune response.

## Long-Term Effects of Preterm Birth on Chronic Inflammatory Diseases

### Increased Risk of Asthma in Preterm Infants

A meta-analysis of 31 birth cohorts showed that preterm birth is associated with an increased risk of wheezing (OR, 1.34 [95% CI, 1.25–1.43]) and school-age asthma (OR, 1.40 [95% CI, 1.18–1.67]) (9). Asthma and wheezing are considered a syndrome comprising multiple phenotypes rather than a single disease (55). The clinical presentations of bronchopulmonary dysplasia (BPD), which affects approximately 10–24% of all VLBW infants in Europe (56) and bronchial asthma overlap. Thus, bronchial hyperreactivity observed in preterm children may be caused by pathophysiological mechanisms other than atopic asthma. This hypothesis is supported by the findings of Siltanen et al., who observed that the incidence of atopy, defined as a positive skin prick test and/or elevated levels of serum IgE, specific IgE, eosinophil cationic protein or eosinophil count, was lower in 10-year-old children who were born as VLBW infants than in term children (25, 57). Moreover, Rosas-Salazar et al. reported that bronchial asthma was frequent in atopic preterm children, but not in non-atopic preterm children (57). The airway hyperresponsiveness was only associated with airway inflammation (defined as elevated fractionated exhaled NO) in atopic preterm children, but not in non-atopic individuals (58).

Preterm neonates are exposed to multiple risk factors for asthma development, for example, cesarean delivery (CD), antibiotic use, and viral infections. Long-term studies are necessary to disentangle the impact of prematurity and the multiple postnatal factors on the risk of asthma development.

There has been a global rise in CD rates from 6.7% in 1990 to 19.1% in 2014 (59). CD significantly alters the immune–microbiota interplay of the newborn and shapes the immunological development.

Cesarean delivery is associated with asthma later in life. A meta-analysis of 23 studies revealed that children born by CD had a 20% higher risk of developing asthma compared with those born vaginally (60). This association was independent of confounding factors such as duration of breast feeding, maternal smoking and low birthweight. In a recent population-based data-linkage study of 321,287 term singleton infants, offspring born by planned CD were at increased risk of asthma (OR, 1.22 [95% CI, 1.11–1.34]) and salbutamol inhaler prescription at age 5 years (OR, 1.13 [95% CI, 1.01–1.26]) as compared with infants born vaginally (61).

Cesarean delivery increases the risk of obesity later in life. Obesity is also a risk factor for asthma. In the US Growing-Up-Today-Study (*n* = 22.068 individuals, 22.3% CD), planned CD was associated with an increased risk of obesity (OR, 1.30 [95% CI, 1.09–1.54]) (62). Pathophysiological links between obesity and asthma are complex and include reduced lung function, increased risk of gastrointestinal reflux, and a common pro-inflammatory state.

Cesarean delivery is associated with gut dysbiosis as a potential mediator for asthma risk (63). Infants born after CD, specifically those born before rupture of the membranes, are colonized with bacteria typical for the skin flora. By contrast, vaginal delivery leads to colonization with bacteria resembling the mother’s rectovaginal flora with predominance of *Lactobacilli* and a higher flora/microbiological diversity (64). In addition, breast milk feeding is often delayed in CD infants hampering the physiological establishment of the microbiome. A pilot study in which infants delivered by CD were exposed to maternal vaginal fluids at birth showed that vaginal microbes can be artificially introduced into the infant’s gut (64). The long-term effects of this approach need to be evaluated.

Cesarean delivery may also have a direct impact on the systemic immune function of neonates. However, assigning a molecular cause–effect relationship is difficult. A piglet model on pre-labor CD showed reduced expression of IFN-γ and a trend toward higher levels of TNF-α as compared with those born vaginally (65). Moreover, CD is associated with decreased monocyte receptor expression (TLR-2 and TLR-4), which is an essential part of innate immunity (66).

These aspects emphasize the idea that early immune–microbiota interaction is a “window of opportunity” in the promotion of long-term health.

Preterm neonates suffer from more viral infections. Respiratory-syncytial virus and rhinovirus, in particular, are associated with a greater risk of recurrent wheeze and asthma (67–69). BPD is an inflammatory disease of the lungs that develops in preterm neonates under the influence of barotrauma, volutrauma, and oxygen toxicity. As a result, the lung develops fibrosis, bronchial hyperreactivity, and an altered microbiome (70). These factors are likely to contribute to the increased susceptibility to viral bronchitis and bronchiolitis (67–69).

Irrespective of any underlying BPD, viral infections are associated with higher morbidity and mortality in premature infants. In retrospectively collected clinical data, Perez et al. showed that very premature children (<32 weeks) had a higher probability of wheezing and a higher frequency of rhinovirus and respiratory-syncytial virus infections in the first 3 years of life, relative to preterm (32–37 weeks) or full-term (>37 weeks) children (71). Moreover, rhinovirus infection in severely premature children was associated with elevated Th2 (IL-4 and IL-13) and Th17 (IL-17) cytokines (72), a pattern that can be also observed in atopic asthma (73).

### Reduced Risk of Atopic Dermatitis in Preterm Neonates

Atopic dermatitis affects 20% of all children in industrialized countries (74). The risk of atopic dermatitis is significantly reduced in preterm neonates (7, 8, 27). Atopic dermatitis is a multifactorial disease, thus the incidence may be lower in preterm neonates for a number of local and systemic reasons.

After birth, transepidermal water loss, stratum corneum hydration, and skin-pH are differentially regulated in various anatomical regions based on the environmental surroundings, which differ from the relatively constant intrauterine exposure to amniotic fluid (75). Moreover, the stratum corneum is formed only weeks after birth (76), and skin surface cytokine levels differ between preterm and term neonates (77). Little is known about the cutaneous immune system in preterm neonates, but it can be hypothesized from studies with disinfectants (78) that transepidermal penetration of environmental antigens is increased in preterm neonates. These factors influence the growth of skin commensals, and consequently, the skin microbiome differs between preterm and term neonates (79, 80).

In addition, systemic factors such as nutrition may influence the risk of atopic dermatitis. Many preterm neonates are not exclusively breast-fed. Prolonged exclusive breast feeding is associated with a higher incidence of atopic dermatitis (81–83). It remains unclear whether partial breast feeding, which is frequently used for preterm neonates, could lower the risk of atopic dermatitis (83).

In summary, preterm birth and early weaning from breast milk are both factors that increase the exposure to a greater variety of antigens, thus reducing the risk of developing atopic dermatitis. Possibly, the premature exposure to a skin microbiome, which differs from mature-born infants, can trigger local immune reactions in preterm neonates that prevent the later development of atopic dermatitis in conjunction with systemic factors.

## Conclusion and Future Directions

Due to care under highly controlled conditions, preterm neonates are a distinct group of patients that can be used as a model to discern (epi-) genetic factors from environmental changes and from maturation-dependent changes in the immune system. Short-term and long-term influences of preterm birth can be measured by comparison to term born children. The influence of preterm birth on the developing immune system is poorly understood but may imprint the risk for immune-mediated diseases later in life (84). Future research should systematically address immunological pathways in the fetus (prenatal), in the preterm neonate and in the mature-born neonate to discern changes that were caused by maturational programs from those that were triggered by premature exposure to the extrauterine environment. The clinical outcome in relation to immune diseases should be assessed, furthering our understanding of the perinatal influences that have a long-term effect on the inflammatory response.

It remains unclear why preterm neonates have a reduced risk of atopic dermatitis and atopy defined as elevated serum IgE, specific IgE, and skin prick test (27). However the increased risk of asthma in preterm neonates is most likely not mediated by an atopic pathophysiology.

The following questions should be addressed in future studies:

(1) Which factors are responsible for the epidemiological differences between asthma and atopic dermatitis in preterm children? In addition to thorough clinical phenotyping and lung function testing, it is essential to include objective analyses for sensitization such as serum IgE, specific IgE, and a skin prick test.
(2) How are the various asthma and atopic dermatitis phenotypes distributed in preterm children?
(3) Is the incidence of autoimmune disease altered in individuals that were born prematurely?
(4) What effect do the microbiome, epigenetics, and other mechanisms have in imprinting the immune system of preterm neonates?

These studies could provide important insights into the mechanisms of immunological imprinting and potential therapeutic interventions to lower the risk of immune-mediated diseases not just in preterm neonates but in the wider population.

## Author Contributions

All authors listed have made a substantial, direct, and intellectual contribution to the work and approved it for publication.

## Conflict of Interest Statement

The authors declare that the research was conducted in the absence of any commercial or financial relationships that could be construed as a potential conflict of interest.
